# Recent developments in vertical transmission of ZIKA virus

**DOI:** 10.18632/oncotarget.11619

**Published:** 2016-08-25

**Authors:** Peter S. Silverstein, Nour A. Aljouda, Anil Kumar

**Affiliations:** Division of Pharmacology and Toxicology, School of Pharmacy, University of Missouri, Kansas City, MO, USA

**Keywords:** ZIKA virus, vertical transmission, placenta, mice, microcephaly

Recently Zika virus (ZIKV) has been the focus of much attention, primarily because of its association with an increased incidence in microcephaly. Although ZIKV was initially discovered in a sentinel rhesus monkey in Uganda [1], its range includes Africa, Southeast Asia, and Latin America. Much of the recent concern regarding ZIKV arises from a dramatic rise in microcephaly that has been reported in Brazil. However, it is only very recently that definitive scientific evidence has demonstrated the ZIKV as cause of microcephaly. In the past few months there have also been several clinical reports regarding the effect of ZIKV infection on pregnant women and their offspring. A recent study in Rio de Janeiro followed 88 pregnant women of whom 72 tested positive for ZIKV in blood and/or urine by RT-PCR [2] and the timing of infection ranged between 5 and 38 weeks of gestation. Doppler ultrasonography on 42 of ZIKA positive and 16 ZIKA negative subjects revealed fetal abnormalities, including microcephaly and ventricular calcification, in 12 of the 42 ZIKA positive (29%) women who underwent this procedure. Conversely, no fetal abnormalities were detected in 16 ZIKA negative women. In a subsequent report, ZIKV was detected and sequenced from amniotic fluid of two of the patients who had fetuses with microcephaly [3]. Thus, detection of the complete viral genome in the amniotic fluid provides evidence that the virus crosses not only the placental barrier, but also causes microcephaly in significant number of pregnancies.

One of the components necessary for the rapid advance of ZIKV research is the availability of a small animal model that faithfully recapitulates the disease process seen in humans. Such a model was developed using mice that lacked type I interferon signaling. Female mice that were Ifnar1−/− were crossed with WT males to produce fetuses that were heterozygous (Ifnar1+/−) [4]. Pregnant dams were inoculated early during embryogenesis (day 6.5 or 7.5) and then sacrificed 7-8 days later. At the time of sacrifice most fetuses had been subject to fetal demise and were reabsorbed. Of those samples that had not undergone fetal demise, viral RNA and infectious virus were detected in the placenta and the fetus, intrauterine growth restriction (IUGR) was also evident and there were apoptotic cells detected in brain samples. Thus, this study established a model which demonstrated in utero transmission of ZIKV along with the pathogenic effects of the virus, such as IUGR and associated damage to the fetal brain. Furthermore, in a model in which WT mice were treated with an anti-ifnar monoclonal antibody the results obtained by the genetic knockout were confirmed, albeit with less severe disease. As with the KO model, ZIKV RNA was detected in both the fetal head and the placenta.

Another recent paper reported the development of a useful mouse model of ZIKV infection and also demonstrated that the virus could be vertically transmitted. The mouse strain that was utilized for infection was WT SJL, rather than a strain defective in immunological response, and the virus used was the Brazilian isolate (ZIKABR) which has been associated with the greatly increased incidence of microcephaly [5]. In this study it was demonstrated that ZIKVBR infection of pregnant SJL mice resulted in progeny which displayed the effects of IUGR along with cortical malformations and detection of viral RNA in the brain. Thus, this was the first direct proof that ZIKVBR could cause birth defects. In addition, using human neural progenitor cells it was shown that ZIKABR is capable of infecting these cells and causing morphological abnormalities as well as increased mortality.

Around the same time, another group demonstrated vertical transmission of ZIKV wherein they infected 10 week old C57 mice either via intra peritoneal or in utero injection. These mice showed infection of radial glia cells of dorsal ventricular zone of fetuses and marked reduction in cortex founder cells [6]. In another paper, published in 18th July issue of Cell Host and Microbe, a group led by Drs. Pereira and Harris showed evidence for two routes (placental and paraplacental) for vertical transmission of ZIKA virus [7].

Taken together these reports demonstrate substantial progress in research focused on ZIKV and microcephaly. The reports described above represent significant advances towards developing appropriate vaccines and/or therapeutics for this affliction. In addition to providing animal models, these studies have provided further evidence for the role of interferons in ZIKV resistance. However, many questions remain. For example, the exact mechanism(s) by which ZIKV causes microcephaly remain to be determined, as does the time during gestation when the fetus is most susceptible to the effects of the virus. Determination of these factors, as well as the elucidation of other aspects of ZIKV-induced microcephaly will facilitate further progress in this research area.
